# Gene signatures for cancer research: A 25-year retrospective and future avenues

**DOI:** 10.1371/journal.pcbi.1012512

**Published:** 2024-10-16

**Authors:** Wei Liu, Huaqin He, Davide Chicco

**Affiliations:** 1 College of Life Sciences, Fujian Agriculture and Forestry University, Fuzhou, China; 2 Dipartimento di Informatica Sistemistica e Comunicazione, Università di Milano-Bicocca, Milan, Italy; 3 Institute of Health Policy Management and Evaluation, University of Toronto, Toronto, Ontario, Canada; University of California San Diego, UNITED STATES OF AMERICA

## Abstract

Over the past two decades, extensive studies, particularly in cancer analysis through large datasets like The Cancer Genome Atlas (TCGA), have aimed at improving patient therapies and precision medicine. However, limited overlap and inconsistencies among gene signatures across different cohorts pose challenges. The dynamic nature of the transcriptome, encompassing diverse RNA species and functional complexities at gene and isoform levels, introduces intricacies, and current gene signatures face reproducibility issues due to the unique transcriptomic landscape of each patient. In this context, discrepancies arising from diverse sequencing technologies, data analysis algorithms, and software tools further hinder consistency. While careful experimental design, analytical strategies, and standardized protocols could enhance reproducibility, future prospects lie in multiomics data integration, machine learning techniques, open science practices, and collaborative efforts. Standardized metrics, quality control measures, and advancements in single-cell RNA-seq will contribute to unbiased gene signature identification. In this perspective article, we outline some thoughts and insights addressing challenges, standardized practices, and advanced methodologies enhancing the reliability of gene signatures in disease transcriptomic research.

## Perspective

### The current scenario on gene signatures

The Cancer Genome Atlas (TCGA) is a comprehensive resource that provides a wealth of genomic and clinical data for various types of cancer [1]. Tens of thousands of “gene signature” scientific papers have been published since the proposal of gene signature by Eric S. Lander in 1999 [2].

**Fig 1:**
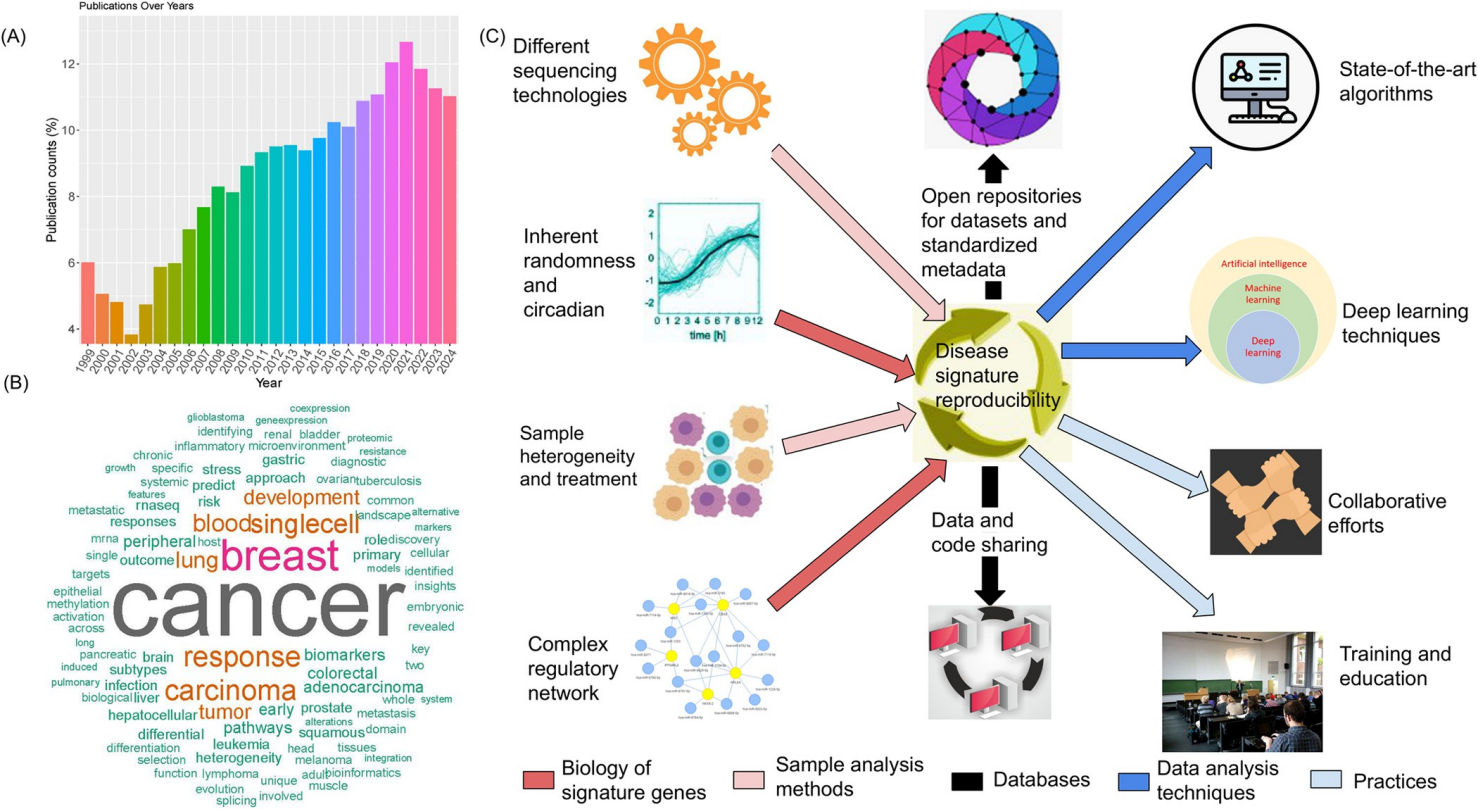
Factors influencing the discovery of new genetic signatures. Thousands of publications about cancer gene signatures have been published in the last 25 years; however, reproducibility is not always possible. (**A**) Publication counts for years 1999–2023. (**B**) Wordcloud shows the frequently mentioned words in publications. (**C**) Factors that can influence and improve the signature reproducibility. The inward arrows indicate factors that may influence the reproducibility of gene signatures, including different sequencing technologies, inherent randomness, sample heterogeneity, and complex regulatory networks. The outward arrows indicate strategies that may help improve the reproducibility of gene signatures, including state-of-the-art algorithms, deep learning techniques, open repositories for datasets and metadata, data and code sharing, collaborative efforts, and training and education.

If one searched “TCGA signature” on Google Scholar, about 90 thousand studies would pop up into the screen (Fig 1A). Currently, TCGA contains multiomics data from 69 primary cancer sites [1], which may mean that each cancer type has been analyzed over 1,000 times. A “gene expression signature” was originally defined as a single gene or a panel of altered genes with validated specificity in terms of diagnosis, prognosis, or prediction of therapeutic response [3]. Over the past 25 years, a large number of gene signatures have been generated for human diseases (Fig 1B) [4]. In 2024, gene expression signatures continue to play a pivotal role in cancer research and clinical applications. These signatures, comprising patterns of gene activities, offer valuable insights into prognosis, treatment response, and patient stratification.

Recent studies, such as those evaluating the PAM50 gene expression signatures in older breast cancer patients [5], underscore their prognostic capacity. Additionally, the Tumor Inflammation Signature (TIS), consisting of 18 genes, developed by Merck in collaboration with NanoString, has demonstrated utility in identifying patients who may benefit from specific cancer treatments [6].

Several gene signatures are clinically available, such as Oncotype and Prosigna [7].

With integrated larger datasets and modern algorithms, better gene expression signature was proposed to improve these commercial platforms [7,8]. Furthermore, advancements like patient-derived gene expression signatures induced by cancer treatment are contributing to a deeper understanding of treatment effects at the molecular level [9].

As technology and research progress, gene expression signatures remain indispensable tools, providing actionable information for personalized medicine and enhancing our comprehension of cancer biology. Key characteristics of those recent gene signatures contain tens of genes that represent the different biological functions of diseases. For example, the 18 genes of TIS cover four areas of biology related to T and natural killer cells, antigen-presenting cells, interferon gamma biology, and T cell exhaustion [6].

Still today, many biological process-related gene signatures have been proposed, such as cuproptosis, necroptosis, ferroptosis, inflammation, epithelial–mesenchymal transition (EMT), interferon gamma–related gene signatures [10–14].

The ultimate aim of gene signatures is to help improve patient therapies and to accelerate precision medicine [15]. However, there are only a small number of overlapping genes across gene signatures, and sometimes even contradictions are observed [16], hindering clinical applications [15]. Similar issues were raised recently in single-cell RNA-seq differential expression analysis [17]. The high rate of false discoveries in single-cell RNA-seq differential expression analysis, as discussed in recent literature [17], poses significant challenges for the clinical application of gene signatures.

The variability and noise intrinsic to single-cell RNA-seq data can lead to differential expression results that are inconsistent with bulk RNA-seq data, which is commonly used in clinical practice. This discrepancy might result in gene signatures that are less reliable and reproducible when applied to heterogeneous tissue samples, thereby limiting their clinical effectiveness.

In this perspective article, we take a snapshot of the overall landscape of gene signatures in bioinformatics, outlining the main challenges and resources within this scientific domain.

### The biology of gene signatures

The inherent dynamic nature of the transcriptome underscores the complexity and continuous fluctuations in the abundance of RNA molecules within a cell or tissue (Fig 1C). Various factors, including cellular composition, tissue origin, and developmental stages, could influence gene expression patterns. Therefore, gene signatures identified from one disease might not work in other diseases.

Gene signatures should not only be functionally annotated but also be bundled with detailed metadata, such as how sampling was performed and which age, sex, and tissue are in the origin cohort [18]. A large fraction of differential gene lists were found to be nonspecific, reflecting shared biology rather than technical artifacts or ascertainment biases [19]. The constant interplay of transcription, RNA processing, and degradation processes adds layers of intricacy to the transcriptomic landscape. Moreover, the diversity of RNA species, such as messenger RNA (mRNA), noncoding RNA (ncRNA), and various splice variants, adds another layer of complexity to the detection process [20].

Moreover, most of current gene signatures are mainly at the gene level, and not at the isoform level. Gene functional complexity includes its diverse roles across tissues, interactions with partners, and multifaceted functions. An example is TGF-β that functions as a tumor suppressor during the initial stages of epithelial carcinogenesis [21].

However, in advanced stages, TGF-β transforms into a tumor promoter, as cancer cells develop resistance to its growth-inhibitory effects through various mechanisms, including alterations in TGF-β signaling components [16]. The impact of TGF-β on cancer cells can be detrimental or beneficial depending on the cellular context [22,23].

However, there are many multifaceted genes within the genome. The current gene signature panel may not consider the effect of combinations of different genes on prognostic performance [24].

It is worth noting that multiple current studies rely on gene signatures from the same biology process. The single biology process-related genes may contain redundancy. An example is the gene coexpression analysis, which focuses on sets of genes but not individual genes and helps to reduce the redundancy [25]. To obtain a better inference of differential gene expression for lowly expressed genes, additional gene coexpression data were integrated to enhance the power of differential analysis [26]. Compared to individual genes, gene coexpression modules have been identified as stable units in cancer cell lines [25]. This may be due to the complexity of gene interactions and gene redundancy.

The dysfunction of different genes may induce the same disease, but the same disease does not always involve dysfunction of the same group of genes. For personalized treatments, a drug that works effectively for a patient might not demonstrate similar efficacy for another patient with the same disease, as the two patients might not share the same dysregulated transcriptome. This aspect might explain why the behavior of a gene signature in one cohort is not always reproducible in another cohort. It has been recognized that each patient is unique [27]: unique life history, unique lifestyle, unique genetics, unique health habits, and so on.

Precision medicine emphasizes tailoring medical care to the specific characteristics of each patient, including genetic makeup, lifestyle, and environmental factors. This approach recognizes that individuals with the same disease may respond differently to treatments based on their unique health profile [28]. Therefore, it is not surprising to observe that gene signature identified in one dataset demonstrates lower accuracy in new datasets [29]. This may partially explain the fact that more than 90% of drug candidates fail during clinical trials, which may take effect only in patients with the exact signature [30].

### Technologies, methods, and best practices

Different sequencing technologies can contribute to discrepancies in the observed gene expression profiles. This variability may arise from differences in sequencing chemistry, sequencing depth, read length, error rates, and other platform-specific features [31]. Consequently, the lack of uniformity in data generation across platforms poses a challenge in achieving consistent and reproducible gene signatures. Researchers need to consider these platform-related factors critically when interpreting and comparing transcriptome data. This issue emphasizes the importance of careful experimental design and analytical strategies to enhance the reliability of findings across different sequencing platforms. New data analysis methods were also developed to perform platform-independent analysis [1–3,32,33].

Variability in gene signatures may be worsened by the utilization of diverse data analysis algorithms and software tools. The choice of different analytical methods, parameter settings, and statistical approaches in fact can introduce significant differences in the interpretation of transcriptomic data: Factors such as normalization techniques [34], differential expression criteria [35], and the handling of batch effects [36,37] contribute to the disparities observed in gene signatures.

Additionally, software-specific features, updates, and algorithmic improvements over time can impact the consistency of results. To enhance the reproducibility of gene signatures, researchers should carefully consider the selection of data analysis tools, adhere to best practices, and conduct robust validation [38–40]. Standardized protocols and benchmarking exercises can aid in evaluating the performance and reliability of different algorithms, ultimately contributing to more consistent and meaningful outcomes in transcriptomic analyses [41–45].

Reproducible gene signature identification faces several challenges, but advancements in methodologies and practices offer promising future perspectives: The establishment of standardized protocols and guidelines for experimental design, sample processing, and data analysis is crucial (Fig 1A). While consensus within the scientific community on best practices will contribute to increased reproducibility [46], continued efforts in benchmarking different algorithms and software tools will provide valuable insights into their strengths and limitations. Comparative studies across multiple platforms and algorithms will aid researchers in making informed choices, promoting transparency and reproducibility, and the integration of multiomics data [47], combining information from genomics, transcriptomics, proteomics, and other layers, can enhance the robustness of gene signature identification [48,49]. ENCODE [50] is an example of a successful multiomics project. Regarding open source software packages, mixOmics [51] in R and INTEGRATE [52] in Python are examples of effective tools for this scope.

This holistic approach might capture a more comprehensive view of molecular changes and increase the reproducibility of identified signatures (Fig 1C).

Moreover, advancements in machine learning and deep learning techniques hold promise for improving the accuracy and reproducibility of gene signature identification [53]. These approaches have the potential to identify complex patterns and interactions within large-scale omics datasets. Embracing open science practices, such as open data sharing, open source code sharing, and transparent reporting, can enhance reproducibility. In this context, the choice of open source programming languages and software packages results being a key pillar of any reliable bioinformatics project: Using open source software code such as R, Python, Rust, or Julia, in fact, can guarantee the free, unrestricted reproducibility of the computational experiments by anyone in the world [54]. Open source popular computational biology projects such as Bioconductor [55], Bioconda [56], and Galaxy [57] deserve special attention for bioinformaticians.

Of course, reproducibility can be impacted by software-specific updates, which might improve the precision of the results but require efforts and energy from researchers to be installed [58,58]. In the just-mentioned platforms (Bioconda, Bioconductor, and Galaxy), these problems are mitigated by special focus and attention on documentation [59].

On the other hand, open repositories for datasets and standardized metadata can facilitate result validation and comparison across studies: Open public online bioinformatics resources can help researchers both find and release new datasets for signature identification.

Some online resources and search engines for open, unrestricted, deidentified biomedical data are the following:

- Gene Expression Omnibus (GEO) [60]
- ArrayExpress [61]
- Sequence Read Archive (SRA) [62]
- Zenodo [63,64]
- Kaggle [65]
- University of California Irvine Machine Learning Repository [66]
- Figshare [67,68]
- PhysioNet [69,70]
- Google Dataset Search [71]
- re3data.org [72]

If data availability is pivotal, also the integration of different data formats is a relevant aspect in this scenario. Collaborative efforts within the scientific community to harmonize data formats, processing pipelines, and analysis workflows and the integration of rigorous quality control measures at various stages of the experimental and analytical processes is pivotal (Fig 1C). Also, standardized metrics for assessing data quality, normalization effectiveness, and batch effect correction can enhance reproducibility, while the utilization of the state-of-the-art single-cell RNA-seq and structured ontology information [73–77] with improved statistical methods can help to identify unbiased gene signatures [78].

Ongoing free training and education initiatives to keep researchers updated on the latest methodologies and best practices, such as Software Carpentry [79], can contribute to forge skilled researchers [80,81]. Moreover, initiatives such as the open access *Education* collection of the *PLOS Computational Biology* journal [82] and the free online bioinformatics video courses on Coursera [83] can be useful for students and researchers worldwide, especially in developing countries.

Biases for reporting genes associated with higher fold changes should be considered [84], and novel algorithms for detecting gene signatures should be developed [85]. Comprehensive and updated gene signature databases should be established, so that researchers can upload their own gene signatures and compare their results with the published data [86], by using different measures that calculate the degree of overlap between gene signatures [4].

Finally, an evidence-based approach is required to translate gene signatures from the laboratory to clinical practice [87]. All these improvements would help to assess the reliability of newly identified gene signatures, which can ultimately influence the discovery of better therapies and drugs, which, in turn, can impact positively the lives of patients in the hospitals (Fig 1C).

In the future, we expect a more frequent adoption of the just-mentioned best practices to discover novel, more robust, and effective gene signatures for cancer research.
